# Data on hypoxia-induced VEGF, leptin and NF-kB p65 expression

**DOI:** 10.1016/j.dib.2018.10.147

**Published:** 2018-11-01

**Authors:** Azizah Al-Anazi, Ranjit Parhar, Soad Saleh, Reem Al-Hijailan, Angela Inglis, Mansour Al-Jufan, Mohammed Bazzi, Sarwar Hashmi, Walter Conca, Kate Collison, Futwan Al-Mohanna

**Affiliations:** a Department of Cell Biology, Riyadh 11211, Saudi Arabia; b Heart Centre, King Faisal Specialist Hospital and Research Centre, Riyadh 11211, Saudi Arabia; c Department of Biochemistry, College of Science, King Saud University, Riyadh 12372, Saudi Arabia; d Developmental Biology, Center for Vector Biology, Rutgers University, New Brunswick, NJ 08901, United States; e Department of Medicine, King Faisal Specialist Hospital and Research Centre, Alfaisal University, Riyadh 11211, Saudi Arabia; f College of Medicine, Alfaisal University, Riyadh 11211, Saudi Arabia

## Abstract

The data set presented here is associated with the article “Intracellular calcium and NF-_k_B regulate hypoxia-induced leptin, VEGF, IL-6 and adiponectin secretion in human adipocytes” (Al-Anazi et al., 2018). Data illustrate hypoxia-induced VEGF and leptin expression in human adipocytes treated with the calcium chelator BAPTA-AM (1 µM). It also shows NF-κB p65 induced expression by hypoxia. Preadipocytes were differentiated for 14 days and then subjected to 0.5–1.5% oxygen in the presence and absence of BAPTA-AM or the NF-κB inhibitor SN50 for 48 h prior to RNA isolation and PCR analysis.

**Specifications table**

**Table:** 

Subject area	*Cell Biology*
More specific subject area	*Adipocyte Biology*
Type of data	*Figure*
How data was acquired	*Differentiation, RT-PCR using CFX96 real-time (RT)-PCR system (Bio-Rad, CA, USA).*
Data format	*Analyzed*
Experimental factors	*Cells were subjected to hypoxia for 48 h in the presence and absence of BAPTA-AM or inhibitor of NF-κB signaling pathway.*
Experimental features	*Cells were lysed and mRNA levels for leptin and VEGF measured*
Data source location	*Department of Cell Biology. King Faisal Specialist Hospital and Research Centre. Riyadh 11211. Saudi Arabia.*
Data accessibility	*Data are with this article*
Related research article	*Al-Anazi A, Parhar R, Saleh S, Al-Hijailan R, Inglis A, Al-Jufan M, Bazzi M, Hashmi S, Conca W, Collison K and Al-Mohanna F. Intracellular calcium and NF-*_*k*_*B regulate hypoxia-induced leptin, VEGF, IL-6 and adiponectin secretion in human adipocytes. 2018. Life Sciences.212:275–284*[1].

**Value of the data**

• The data can be used to show that lowering intracellular calcium concentrations selectively increase adipocyte expression of VEGF and leptin *in vitro*.
• NF-kB p65 expression is induced by hypoxia.
• Data can be used to show the inhibition of hypoxia-induced NF-kB p65 expression by SN50 in adipocytes.

## 1 Data

**Fig. 1:**
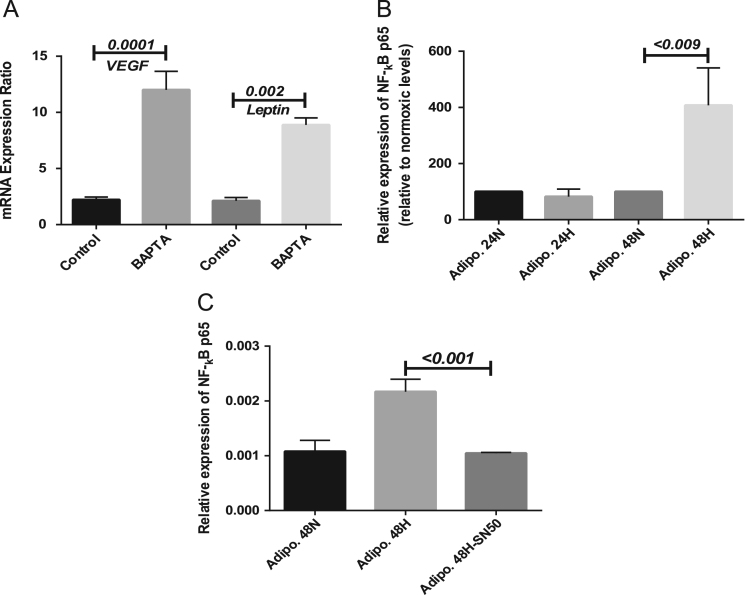
Hypoxia-induced (48 h) expression of VEGF and leptin is augmented by BAPTA-AM (1 µM). Data are expressed as mean ± SEM; *n* ≥ 3 performed in triplicate, and are representative of at least three independent experiments. mRNA is expressed relative to 18S ribosomal RNA. Sidak׳s corrected multiple comparison *p*-values are indicated on the black horizontal bars (A). (B) Hypoxia-induced NF-_**k**_B p65 expression relative to normoxic levels. (C) Inhibition of NF-_**k**_B p65 expression by SN50 (20 µM). mRNA is expressed relative to 18S ribosomal RNA. Sidak׳s corrected multiple comparison *p*-values are indicated on the black horizontal bars. Data are mean ± SEM *n* ≥ 3 performed in triplicate and are representative of at least three independent experiments.

Expression of VEGF and leptin in the presence of BAPTA-AM under hypoxic conditions, the expression of NF-kB p65 under normoxic and hypoxic conditions and the inhibition of hypoxia-induced expression of NF-kB by SN50 (Fig. 1). For details, please see our full article [1].

## 2 Experimental design, materials, and methods

Human subcutaneous preadipocytes (HPAd) were grown in preadipocyte growth medium (PGM). Preadipocyte were treated with adipocyte differentiation medium (ADM) containing PGM supplemented with SingleQuots™ (containing insulin, dexamethasone, indomethacin, and isobutyl-methylxanthine) for 14 days. Adipocyte differentiation was monitored by the appearance of lipid droplets (typically commencing on days 4–5 post initiation). Hypoxia was induced by adipocyte exposure to 0.5–1.0% oxygen in hypoxia chamber (Biospherix, Ltd, Parish, NY, USA) for 48 h. Parallel experiments with control cells were performed (under 21% oxygen). For calcium chelation, experiments were performed in the presence or absence of the calcium chelator (BAPTA-AM, 1 µM) during exposure to hypoxia. NF-κB inhibition was achieved by treating the cells with the NF-κB inhibitor SN50 (20 µM). Total cellular RNA and protein was extracted from the cells and stored for subsequent analysis.

## References

[1] Al-Anazi A., Parhar R., Saleh S., Al-Hijailan R., Inglis A., Al-Jufan M., Bazzi M., Hashmi S., Conca W., Collison K., Al-Mohanna F. (2018). Intracellular calcium and NF-_k_B regulate hypoxia-induced leptin, VEGF, IL-6 and adiponectin secretion in human adipocytes. Life Sci..

