# Supplementary material for: Data on hypoxia-induced VEGF, leptin and NF-kB p65 expression
Source: Data Brief. 2018 Nov 1;21:2395–7. doi: 10.1016/j.dib.2018.10.147 (PMC6282628; doi:10.1016/j.dib.2018.10.147)
Supplement: Supplementary file 1 — Supplementary material [file mmc1.pdf]

ELSEVIER

Elsevier

Radarweg 29

Amsterdam 1043 NX,

elsevier.com

Empowering Knowledge

Life Sciences

Conflict of Interest Policy

Article Title:

Intracellular calcium and NF-kB regulate hypoxia-induced leptin, VEGF, IL-6 and adiponectin secretion in human adipocytes

Author Names:

Azizah Al-Anazi1, Ranjit Parhar1, Soad Saleh1, Reem Al-Hijailan1, Angela Inglis1, Mansour Al-Jufan2, Mohammed Bazzi3, Sarwar Hashmi4, Walter Conca1,5,6, Kate Collison1 and Futwan Al-Mohanna1,6

Declarations

Life Sciences require that the **corresponding author**, signs on behalf of all authors, a declaration of conflicting interests. If you have nothing to declare in any of these categories then this should be stated.

Conflict of Interest

A conflicting interest exists when professional judgment concerning a primary interest (such as patient's welfare or the validity of research) may be influenced by a secondary interest (such as financial gain or personal rivalry). It may arise for the authors when they have financial interest that may influence their interpretation of their results or those of others. Examples of potential conflicts of interest include employment, consultancies, stock ownership, honoraria, paid expert testimony, patent applications/registrations, and grants or other funding.

Please state any competing interests:

The authors declare no conflict of interest

Funding Source

All sources of funding should also be acknowledged and you should declare any involvement of study sponsors in the study design; collection, analysis and interpretation of data; the writing of the manuscript; the decision to submit the manuscript for publication. If the study sponsors had no such involvement, this should be stated.

Please state any sources of funding for your research:

The work was partially supported by King Abdulaziz City for Science and Technology grant number KACST 0968-12 and KFSHRC research funds

Elsevier
Radarweg 29
Amsterdam 1043 NX,
elsevier.com

Empowering Knowledge

Author Contribution to Study

All authors listed on your paper must have made significant contributions to the study. To ensure clarity, you are required to enter the specific details of each author's contribution, which must substantiate the inclusion of each person on the manuscript. Please detail this information below (submit additional sheets as necessary):

Author Name	Author Email	Specific Role in Study
Azizah Al-Anazi	aenizi@kfshrc.edu.sa	Performed experiments
Ranjit Parhar	dr.parhar@gmail.com	Instigated and performed experiments
Soad Saleh	ssaleh@kfshrc.edu.sa	Performed experiments
Reem Al-Hijailan	rhijailan@kfshrc.edu.sa	Performed experiments
Angela Inglis	ainglis@kfshrc.edu.sa	Performed experiments
Mansour Al-Jufan	maljufan@kfshrc.edu.sa	Instigated ideas and writing
Mohammed Bazzi	mbazzi@ksu.edu.sa	Helped in writing

Signature

Print name

Futwan Al-Mohanna PhD. FRSB. FRSC.

Elsevier
Radarweg 29
Amsterdam 1043 NX,
elsevier.com

Empowering Knowledge

Author Contribution to Study

All authors listed on your paper must have made significant contributions to the study. To ensure clarity, you are required to enter the specific details of each author's contribution, which must substantiate the inclusion of each person on the manuscript. Please detail this information below (submit additional sheets as necessary):

Author Name	Author Email	Specific Role in Study
Sarwar Hashmi	sarwar.hashmi@rutgers.edu	Writing and correction
Walter Conca	wconca@kfshrc.edu.sa	Writing and correction
Kate Collison	kate@kfshrc.edu.sa	Instigation of ideas, writing and correction
Futwan Al-Mohanna	futwan@kfshrc.edu.sa	Wrote the paper and supervised the team.

Signature

Print name

Futwan Al-Mohanna PhD. FRSB. FRSC.
